# Minimal Clinically Important Differences in Gait and Balance Ability in Patients Who Underwent Corrective Long Spinal Fusion for Adult Spinal Deformity

**DOI:** 10.3390/jcm12206500

**Published:** 2023-10-12

**Authors:** Tomoyoshi Sakaguchi, Umesh Meena, Masato Tanaka, Hongfei Xiang, Yoshihiro Fujiwara, Shinya Arataki, Takuya Taoka, Kazuhiko Takamatsu, Yosuke Yasuda, Masami Nakagawa, Kayo Utsunomiya

**Affiliations:** 1 Department of Rehabilitation, Okayama Rosai Hospital, 1-10-25 Chikkomidorimachi, Minami Ward Okayama, Okayama 702-8055, Japan; tomoyoshi0127@gmail.com (T.S.); kazuhikopt0803@gmail.com (K.T.); kyushudanji19861007@gmail.com (Y.Y.); ot2632nakagawa@gmail.com (M.N.); www.kayo68@gmail.com (K.U.); 2 Department of Orthopedic Surgery, Okayama Rosai Hospital, 1-10-25 Chikkomidorimachi, Minami Ward Okayama, Okayama 702-8055, Japan; gtbumesh@gmail.com (U.M.); xianghf@qdu.edu.cn (H.X.); fujiwarayoshihiro2004@yahoo.co.jp (Y.F.); araoyc@gmail.com (S.A.); taokatakuya@gmail.com (T.T.)

**Keywords:** adult spinal deformity, surgery, rehabilitation, spinal balance, minimal clinically important difference (MCID)

## Abstract

Study Design: Retrospective observational study. Background: The risk of a femoral neck fracture due to a fall after adult spinal deformity surgery has been reported. One of the most significant factors among walking and balance tests in post-operative ASD patients was the timed up-and-go test (TUG). This study aims to calculate the minimal clinically important difference (MCID) in balance tests after ASD surgery. Methods: Forty-eight patients, 4 males and 44 females, were included by exclusion criteria in 66 consecutive patients who underwent corrective surgery as a treatment for ASD at our institution from June 2017 to February 2022. The inclusion criteria for this study were age ≥50 years; and no history of high-energy trauma. The exclusion criteria were dementia, severe deformity of the lower extremities, severe knee or hip osteoarthritis, history of central nervous system disorders, cancer, and motor severe paralysis leading to gait disorders. The surgeries were performed in two stages, first, the oblique lumber interbody fusion (OLIF) L1 to L5 (or S1), and second, the posterior corrective fusion basically from T10 to pelvis. For outcome assessment, 10 m walk velocity, TUG, ODI, and spinopelvic parameters were used. Results: Ten meter walk velocity of pre-operation and post-operation were 1.0 ± 0.3 m/s and 1.2 ± 0.2 m/s, respectively (*p* < 0.01). The TUG of pre-operation and post-operation were 12.1 ± 3.7 s and 9.7 ± 2.2 s, respectively (*p* < 0.01). The ODI improved from 38.6 ± 12.8% to 24.2 ± 15.9% after surgery (*p* < 0.01). All post-operative parameters except PI obtained statistically significant improvement after surgery. Conclusions: This is the first report of MCID of the 10 m walk velocity and TUG after ASD surgery. Ten meter walk velocity and the TUG improved after surgery; their improvement values were correlated with the ODI. MCID using the anchor-based approach for 10 m walk velocity and the TUG were 0.10 m/s and 2.0 s, respectively. These MCID values may be useful for rehabilitation after ASD surgery.

## 1. Introduction

Approximately 1/3 of the population are affected by adult spinal deformity (ASD) [1]. ASD is a complex disease that causes low back pain, activity of daily life (ADL) disturbance, and spinal balance problems in the elderly population [2,3]. When conservative treatment is not effective, surgical intervention is indicated, and its efficacy has been reported [4]. The surgery aims to correct and prevent the progression of the deformity, relieve pain, and decompress the associated stenotic spinal canal [5]. Elderly ASD patients find it immensely difficult to adapt to the new posture after surgery [6]. Physical therapy is very important to regain spinal balance after ASD corrective surgery so the patients can avoid accidental falls due to spinal imbalance [7]. The risk of a femoral neck fracture due to falls after ASD surgery has been reported [8]. A systematic review concluded that complex physical therapy following deformity correction surgery reduces fear avoidance behavior [9]. A significant factor among walking and balance tests in post-operative ASD patients was the timed up-and-go test (TUG) which correlated with the Oswestry disability index (ODI) after ASD surgery [10,11].

The term minimal clinically important difference (MCID) was first described by Jaeschke and colleagues in 1989 [12]. Although statistically significant changes often occurred during the use of instruments that measured change after intervention, in some cases, the significant change had little clinical significance. The definition of MICD is the smallest difference in value t, which patients perceive as beneficial and which would mandate, in the absence of troublesome side effects and excessive cost, a change in the patient’s management [12]. MICD is recognized as an approach to elucidating the significance of changes in scores in quality-of-life instruments by comparing them [12]. Understandably, ASD patients below MICD lack the potential to reach significant clinical improvements in terms of low back pain. This MCID was used to evaluate gait and balance after lumber degenerative disc disease and lumber spinal canal stenosis [9,13]. However, there is no report of MCID after ASD surgery. This study aims to calculate the MCID of the balance test after ASD surgery.

## 2. Materials and Methods

**Figure 1:**
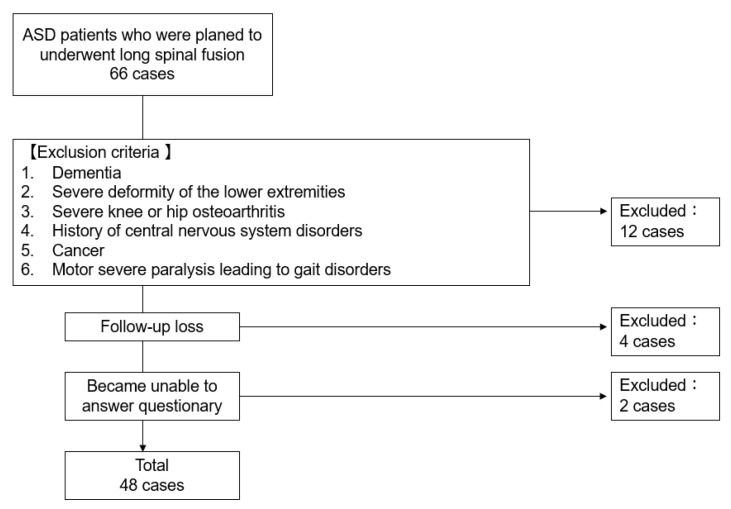
Patient selection.

Forty-eight patients, 4 males and 44 females, were included by exclusion criteria in 66 consecutive patients who underwent corrective surgery as a treatment for ASD at our institution from June 2017 to February 2022 (Figure 1). The inclusion criteria were age over 50 years or older and who showed radiographical evidence of at least one of the following: sagittal vertical axis (SVA) of 95 mm or more, pelvic tilt (PT) of 30 degrees or greater, and/or coronal Cobb angle of 30 degrees or greater [14]. Exclusion criteria were dementia, severe deformity of the lower extremities, severe knee or hip osteoarthritis, history of central nervous system disorders, cancer, and motor severe paralysis leading to gait disorders.

The surgeries were performed in two stages, first, oblique lumber interbody fusion (OLIF) L1 to L5 (or S1), and second, posterior corrective fusion basically from T10 to pelvis. The timed analysis has been acceded to by the review board of our establishment (No. 417).

### 2.1. Patient Demographics

For patient factors, age of surgery, gender, height, weight, BMI, and comorbidities, such as diabetes mellitus (DM) and cerebral infarction, were evaluated (Table 1). For surgery-related factors, surgical time, intraoperative blood loss, and fusion level were investigated (Table 2).

### 2.2. Outcome Assessment

#### 2.2.1. Ten Meter Walk Velocity

**Figure 2:**
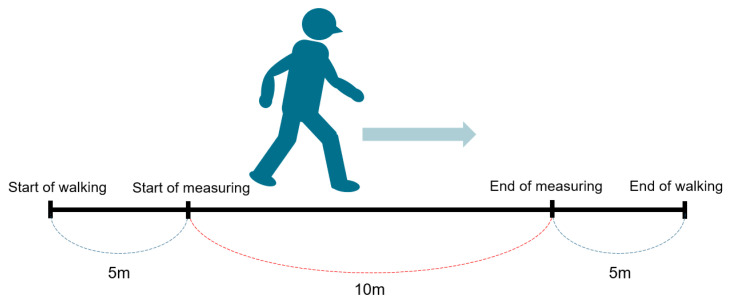
Ten meter walk velocity.

The 10 m walk test is a performance measure used to assess walking speed in meters per second over a short distance [15]. It can be employed to determine functional mobility, gait, and vestibular function. Normative values are 1.34–1.24 m/s for 60–69 years old, and 1.26–1.13 for 70–79 years old [16]. Equipment required a stopwatch and a clear pathway with a set 16 m distance. The examiner measures and marks a clear path of at least 10 m in length and adds a mark at 3 m at both ends (Figure 2). The total time taken to ambulate 10 m is recorded. Timing starts when the toes pass the 3 m mark and timing stops when the toes pass the 13 m mark. The 10 m is then divided by the total time taken (in seconds) to complete and the total time is recorded in m/s.

The examiner instructs as follows: the individual walks without assistance for 20 m, with the time measured for the intermediate 10 m to allow for acceleration and deceleration. Start timing when the toes pass the 5 m mark. Stop timing when the toes pass the 15 m mark. Perform two trials and calculate the average of three trials. Patients are instructed by the examiner “I will say ready, set, go. When I say go, walk at your normal comfortable speed until I say stop”. The 10 m walk test has demonstrated excellent reliability in many conditions, including healthy adults, Parkinson’s disease, hip fractures, and spinal cord injury. For healthy adults, the test-retest reliability for comfortable gait speed (r = 0.75–0.90) and excellent interrater reliability (ICC = 0.98) [17].

#### 2.2.2. Timed Up-and-Go Test (TUG)

**Figure 3:**
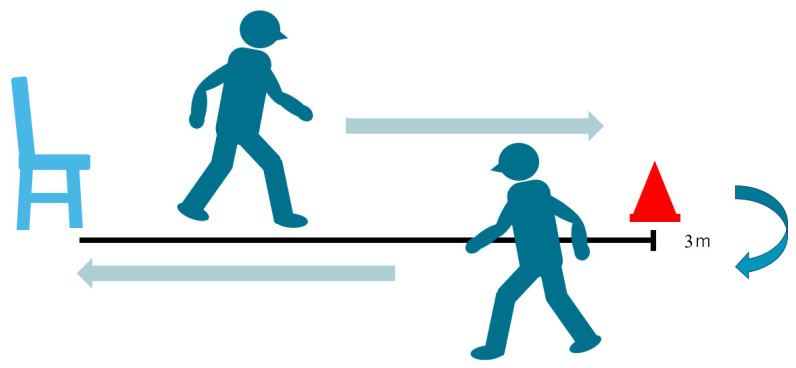
Timed Up-and-Go Test (TUG).

The timed up-and-go test (TUG) is used to assess mobility and dynamic balance [18] (Figure 3). The examiner uses a stopwatch to measure the time. Patients wear their regular footwear and can use a walking aid if needed. Begin by having the patient sit back in a standard armchair and identify a line 3 m or 10 feet away, on the floor. First, the examiner instructs the patient when I say “Go”, I want you to stand up from the chair, walk to the line on the floor at your normal pace, then turn, walk back to the chair at your normal pace, and finally sit down again. The examiner should always stay by the patient for safety.

The word “Go” is the beginning timing, and then the examiner stops timing after the patient sits back down and records the time. In this study, the patient asked for this test twice and the better time was adopted. An older adult who takes ≥12 s to complete the TUG is at risk for falling. The average values are 60–69 years old; 7.9 ± 0.9 s, 70–79 years old; and 7.7 ± 2.3 s [19]. For healthy adults, the TUG has demonstrated excellent interrater reliability (ICC = 0.99) [20] and intra-rater reliability (ICC = 0.99) [21] in healthy older adults.

The excellent reliability and validity of the TUG as a clinical tool have been reported in a systematic review [22].

#### 2.2.3. Patients Reported Outcomes (PRO)

The Japanese version of the ODI, a lumbar spine-specific ADL evaluation method, was used [23]. MCID of the ODI in ASD surgery was reported as 11% [24].

### 2.3. Radiographic Measurements

**Figure 4:**
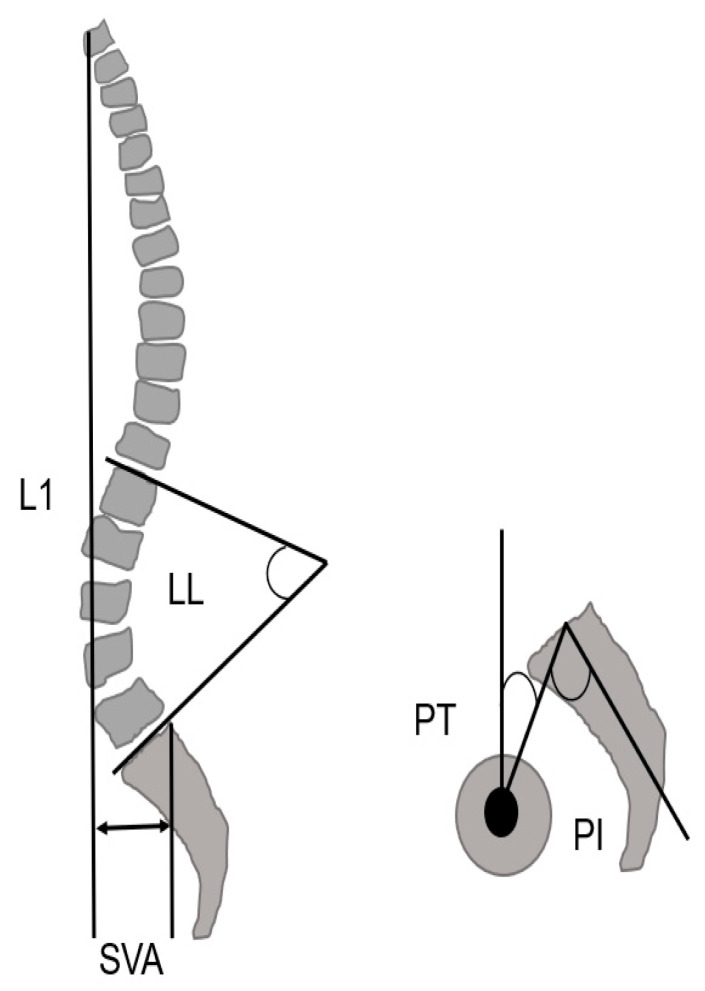
Spinopelvic parameter, SVA; sagittal vertical axis, LL; lumbar lordosis, PT; pelvic tilt, PI; pelvic incidence (cited from [10]).

**Figure 5:**
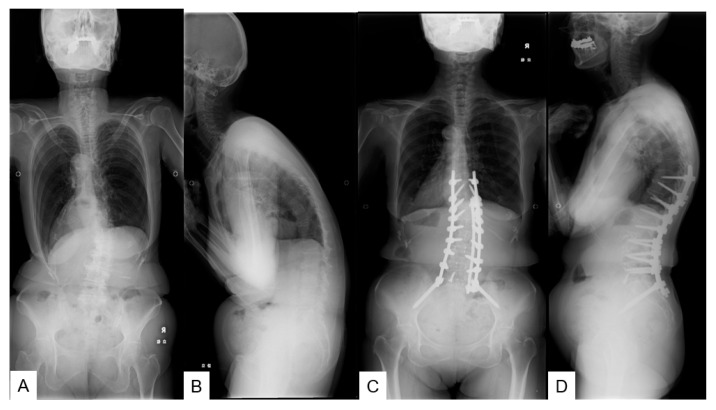
A 72-years-old female with adult spinal deformity, L1-S1 OLIF, and T10-pelvis posterior fusion. (**A**): Pre-operative posteroanterior radiogram, (**B**): Pre-operative lateral radiogram, (**C**): Post-operative posteroanterior radiogram, (**D**): Post-operative lateral radiogram.

The radiographic parameters of the spine and pelvis (lumbar lordosis; LL, pelvic tilt; PT, sagittal vertical axis; SVA, pelvic incidence; PI) were measured pre-operatively and 12 months post-operatively (Figure 4 and Figure 5) [25].

### 2.4. Statistical Analysis

Internal responsiveness was compared using either a paired-*t* test or a Wilcoxon signed-rank test for changes in 10 m walk velocity, TUG, and ODI before and 1 year after ASD surgery. External responsiveness was evaluated using an anchor-based approach [26]. The ODI was used for anchor. The relationship between 10 m walk velocity, TUG, and change in the ODI was evaluated using the Spearman Rank Correlation Coefficient. The Receiver Operating Characteristic curve (ROC curve) was used to discriminate between responders and non-responders, and the cutoff values, sensitivity, specificity, and area under the curve for 10 m walk velocity and the TUG were examined.

The Mann–Whitney U test was used to compare pre-operative measures in responders and non-responders. A paired-*t* test was used to analyze spinopelvic parameter change before and after surgeries. The software utilized to process the data was EZR version 1.61 [27] and *p* < 0.05 was noted as remarkably significant. All numerical values of the cohort expressed are as mean ± standard deviation (SD).

## 3. Results

### 3.1. Chronological Results of 10 m Walk Velocity and TUG (Figure 6 and Figure 7)

**Figure 6:**
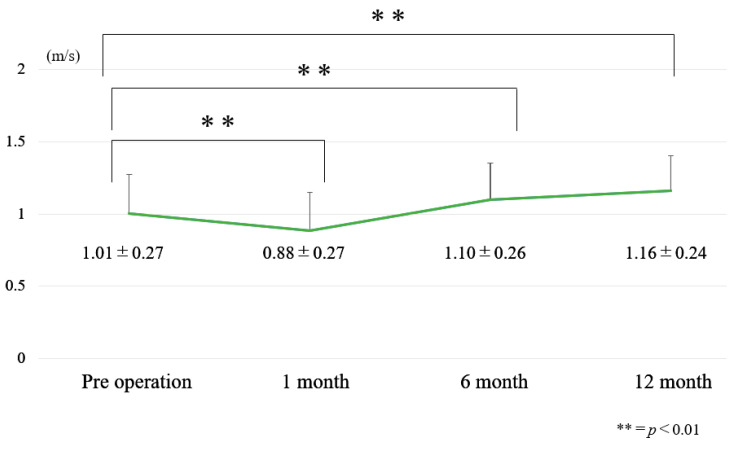
Chronological results of 10 m velocity.

**Figure 7:**
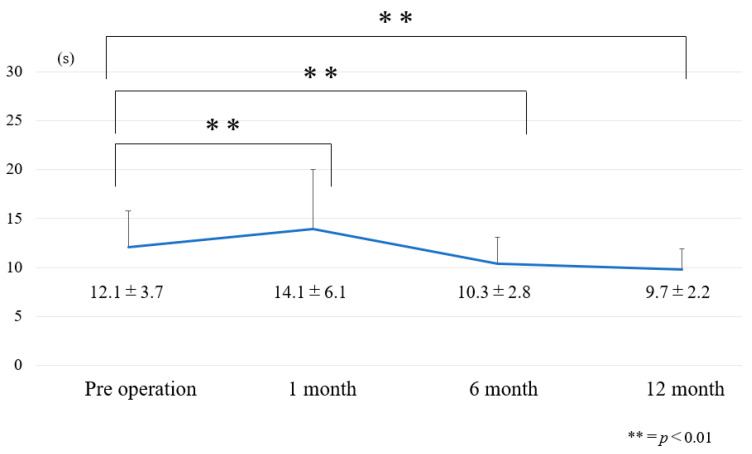
Chronological results of TUG.

10 m velocity became slower at one month postoperatively and became faster at 6 months postoperatively (Figure 6). At one month after surgery, dynamic spinal balance (TUG) became worse. However, TUG became better at 6 months and 12 months after surgery (Figure 7). 

### 3.2. Internal Responsiveness

The 10 m walk velocity of pre-operation and post-operation (one year) were 1.0 ± 0.3 m/s and 1.2 ± 0.2 m/s, respectively (*p* < 0.01). The TUG of pre-operation and post-operation were 12.1 ± 3.7 s and 9.7 ± 2.2 s, respectively (*p* < 0.01). The ODI was improved from 38.6 ± 12.8% to 24.2 ± 15.9% after surgery (*p* < 0.01). Each effect sizes were 0.63, 0.78, and 1.0 (Table 3).

### 3.3. External Responsiveness

#### 3.3.1. Anchor-Based Approach

There were 30 ODI responders and 18 non-responders. There was no statistical difference between open and MIS surgery. The amount of intraoperative blood loss did not affect surgical complications, such as epidural hematoma or neurological deterioration. Furthermore, this is not related to the results of spinal balance or walk velocity.

**Figure 8:**
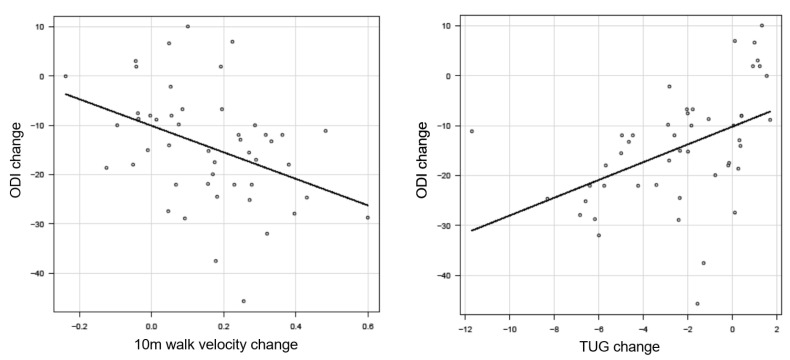
ROC analysis of 10 m walk velocity and TUG.

**Figure 9:**
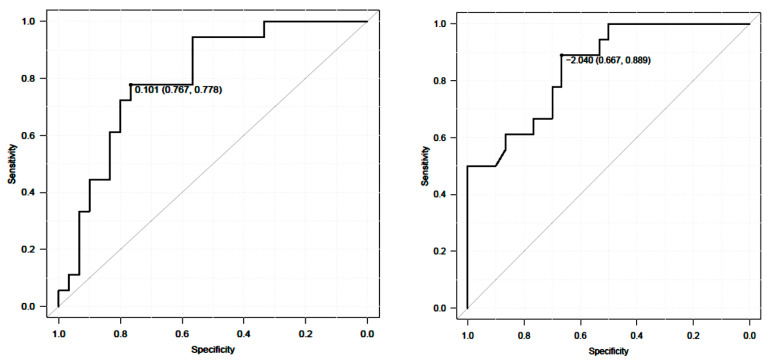
Sensitivity and specificity of 10 m walk velocity and TUG.

External responders of 10 m walk velocity and the TUG were referenced in Table 4 and Table 5. There was a significant correlation between 10 m walk velocity and ODI (r = −0.41, *p* < 0.01) (Figure 8). We recognized a meaningful correlation between the TUG and ODI (r = 0.57, *p* < 0.01) (Figure 9).

The ROC analysis prevailed cutoff value of 10 m walk velocity was 0.10 m/s. The AUC was 0.8% and the 95% CI was 0.67~0.93 (sensitivity 0.78, specificity 0.77) (Figure 8). A cutoff value of TUG was −2.04 s. The AUC was 0.85%, and the 95% CI was 0.74~0.96 (sensitivity 0.67, specificity 0.85) (Figure 9).

#### 3.3.2. Post-Operative Comparison of Responders and Non-Responders

There was no statistical difference in all spinopelvic parameters between responders and non-responders (Table 6). Responders were defined as an improvement of ODI by more than 11% [24].

#### 3.3.3. Spinopelvic Parameter

All post-operative parameters except PI showed statistically significant improvement after surgery (Table 7).

## 4. Discussion

Adult spinal deformity (ASD) is caused by spinal malalignment, which results in severe low back pain, neurological dysfunction, and severe deformity of the body. One of the most severe signs of ASD is gait abnormalities. ASD patients’ spinal balance can be checked using spinopelvic parameters, which are crucial and helpful indicators [7]. Gait posture and standing posture, however, alter over a brief period [28]. Spinal deformity worsens the patient’s dynamic balance and increases the incidence of fall [29,30]. Accordingly, the patient who falls has a higher risk of a hip fracture, which then deteriorates the patient’s quality of life [31].

The 10 m walk test is a performance indicator that measures walking speed over a short distance in meters per second. It can be used to assess functional mobility, gait, and vestibular function. The 10 m walk test is an easy and effective test to look for actual gait disruption. Spinal balance is crucial for elderly patients to avoid falls while walking [7]. Elderly patients who fall may sustain hip fractures that worsen their ambulatory performance and increase their need for assistance with daily activities. The TUG, on the other hand, is a quick test that evaluates mobility and calls for both static and dynamic balance. This examination is typically carried out to determine whether the patient can safely venture outside on their own [22]. Pre-operative data from these tests showed that ASD patients performed worse, suggesting that they may be more likely to experience falls and gait disturbances. Yagi et al. reported that, in comparison to healthy individuals, ASD patients had an asymmetrical gait pattern and impaired gait ability [32]. In this study, after surgery, all data improved over the pre-operative status. The results of the 10 m walk velocity pre- and post-operation were 1.0 ± 0.3 m/s and 1.2 0.2 m/s, respectively. The result of a 10 m walking speed test, according to Tomoyoshi et al. [10], was 1.09 ± 0.25 m/s. This result is higher than 1.00 m/s, which denotes the typical pace of stable walking. The TUG of pre-operation and post-operation were 12.1 ± 3.7 s and 9.7 ± 2.2 s, respectively. The ODI was improved from 38.6 ± 12.8% to 24.2 ± 15.9% after surgery. There was a significant correlation between 10 m walk velocity and ODI and TUG. Tomoyoshi et al. [10] observed that there is a significant link between the TUG and 10 m walking speed, which is identical to our data. All post-operative parameters, except PI, obtained a statistically significant improvement after surgery.

In the analysis of the randomized cohort, operative treatment was associated with greater improvement at the 2-year follow-up in the SRS-22 and the ODI than conservative treatment, MCID in the SRS-22 (85.7% versus 38.7%; *p* < 0.001), and the ODI (77.4% versus 38.3%; *p* < 0.001) [33]. In our study, 62.5% of patients obtained more than the MCID. In this study, the MCID for the 10 m walk speed and the TUG were 0.1 m/s and 2.0 s, respectively. There were several MCIDs for walk speed, and TUG reported spinal illnesses and rehabilitation being conserved [6,34]. For older individuals receiving therapy, the MCID of walk speed was 0.08 m/s [34]. For patients with chronic low back pain, the MCID of TUG following spinal fusion was 1.3 s [6]. After a protracted spinal fusion, ASD patients exhibit altered proprioception, sensorimotor integration failure, and postural reflex dysfunction [6]. Furthermore, compared to the general population, patients’ walking ability declines after surgery [26]. Our findings indicate that ASD patients must receive more MCID post-operatively than individuals with other spinal diseases. Rehabilitation boosted the patients’ capacity to walk and the muscle strength in their legs following surgery, which improved their quality of life [35]. Pre-operative rehabilitation is crucial for this type of patient as well, in our opinion. The TUG is said to be improved by exercising the trunk muscles [36]. After corrective long spinal fusion for ASD, muscle powers of the hip flexor and knee extensor were reduced then patient’s gait ability and spinal balance became worse. Rehabilitation such as muscle exercise for these muscles is necessary after surgery [37]. Disuse atrophy of the trunk muscles is seen in ASD patients [38]. Exercise of the trunk muscles to enhance dynamic balance is the main component of pre-operative rehabilitation. Post-operative rehabilitation then works better. For evaluating ASD surgery, it is helpful to utilize the MCID of 10 m walk velocity as 0.10 m/s and the TUG as 2.0 s. The rehabilitation team might use these values to examine prognostic factors or to focus on the patients’ goals following ASD surgery. The results of this study contribute both clinical application and research. In clinical aspect, this MCID is useful for the set point of early post-operative physical therapy interventions targeting physical activity. Furthermore, this is used as effect measurement of some therapeutic intervention [39]. In research aspect, this MICD enables the retrospective study about important factors to achieve this MICD in rehabilitation after ASD surgery [40,41].

### Study Limitations

There were several limitations to this study. The sample size was small and there was gender inequality. This study included only Japanese patients and it is not a multicenter study.

## 5. Conclusions

This is the first report of MCID of 10 m walk velocity and the TUG after ASD surgery. Ten meter walk velocity and the TUG improved after surgery and their improvement values were correlated with the ODI. MCID using the anchor-based approach of 10 m walk velocity and the TUG were 0.10 m/s and 2.0 s, respectively. These MCID values may be useful for rehabilitation after ASD surgery.

## Figures and Tables

**Table 1:** Patient demographics.

	*n* = 48
Gender (Men:Women)	4:44
Age (mean ± S.D.) (year)	71.0 ± 7.4
Height (mean ± S.D.) (cm)	151.0 ± 7.7
Body weight (mean ± S.D.) (kg)	52.8 ± 9.9
Body mass index (mean ± S.D.) (kg/m^2^)	23.2 ± 3.7
Comorbidity	13 (27.1%)
Heart disease	5 (10.4%)
Diabetes mellitus	4 (8.3%)
Depression	4 (8.3%)

**Table 2:** Surgery-related factors.

	*n* = 48
**Operation time (min)**	
1st stage (OLIF)	200.2 ± 47.8
2nd stage (posterior corrective fusion)	280.5 ± 53.5
**Bleeding (mL)**	
Blood loss at OLIF surgery (mL)	437.4 ± 317.1
Blood loss at posterior surgery (mL)	824.3 ± 385.3
Type of Posterior fusion (OPEN/MIS)	30/18
Upper instrumented vertebra (UIV)	T6: 2, T9: 2, T10: 44

**Table 3:** Pre-operative and post-operative one-year value of 10 m walk velocity, TUG, and ODI.

	10 m Walk Velocity	TUG	ODI
Pre-operative	1.0 ± 0.3	12.1 ± 3.7	38.6 ± 12.8
Post-operative 1 year	1.2 ± 0.3	9.7 ± 2.2	24.2 ± 15.9
Amount of change	0.2 ± 0.2	−2.3 ± 3	−14.4 ± 11.6
*p* value	<0.01	<0.01	<0.01
Effect size	0.63	0.78	1.0

**Table 4:** External responsiveness of 10 m walk velocity: Anchor-based approach.

10 m Walk Velocity	Change ODI (*n* = 30)
Anchor-Based Approach	
Average change 10 m walk velocity	0.22 ± 0.2
Change difference 10 m walk velocity	0.18
ROC analysis	
AUC (95% CI)	0.8
Cutoff value	0.10
Sensitivity	0.78
Specificity	0.77

ROC: receiver operating characteristic, AUC: area under the curve.

**Table 5:** External responsiveness of TUG: Anchor-based approach.

Timed Up and Go Test	Change ODI (*n* = 30)
Anchor-Based Approach	
Average change 10 m walk velocity	−3.6 ± 2.9
Change difference 10 m walk velocity	−3.3
ROC analysis	
AUC (95% CI)	0.85
Cutoff value	−2.04
Sensitivity	0.89
Specificity	0.67

ROC: receiver operating characteristic, AUC: area under the curve.

**Table 6:** Post-operative comparison of responders and non-responders.

	Responders (*n* = 30)	Non Responders (*n* = 18)	*p* Value
Age at surgery (year)	70.9 ± 8.1	72.6 ± 6.4	0.53
Sex (male/female)	4/26	0/18	0.17
Body Mass Index (kg/m^2^)	22.9 ± 3.4	23.5 ± 4.3	0.94
10 m walk velocity (m/s)	0.98 ± 0.3	1.04 ± 0.2	0.25
TUG (s)	12.9 ± 4.3	10.6 ± 1.7	0.12
Oswestry disability index (%)	39.2 ± 12.8	37.8 ± 12.4	0.59
Sagittal vertical axis (mm)	108 ± 46.1	106.4 ± 44.7	0.72
Lumbar lordosis (degree)	14.1 ± 17.8	7.7 ± 0.3	0.17
Pelvic tilt (degree)	34.4 ± 9.9	34.2 ± 7.2	0.96
Pelvic incidence (degree)	53 ± 7.9	54.1 ± 7.2	0.71
PI-LL (degree)	41.5 ± 19.1	43.4 ± 11.9	0.69

PI-LL: Pelvic incidence-Lumbar lordosis.

**Table 7:** Pre-operative and post-operative spine and pelvic parameters.

	Preoperation	Postoperation	*p* Value
Sagittal vertical axis (mm)	107.4 ± 46.1	38.3 ± 32.3	<0.001
Lumbar lordosis (degree)	11.6 ± 15.8	45.7 ± 9.6	<0.001
Pelvic tilt (degree)	34.3 ± 9.1	19.6 ± 9.8	<0.001
Pelvic incidence (degree)	52.3 ± 6.8	52.1 ± 7.2	0.19
PI-LL (degree)	41.7 ± 16.6	7.71 ± 12.3	<0.001

PI-LL: Pelvic incidence-Lumbar lordosis.

## Data Availability

The data presented in this study are available in the article.
